# Quercetin Protects against Ethanol-induced Neurodegeneration in Adult Rat Cortex

**DOI:** 10.2174/011570159X349349250118152027

**Published:** 2025-05-29

**Authors:** Haroon Badshah, Rehman Zafar, Heeyoung Kang, Sangbae Ma, Haroon Khan, Myeong Ok Kim

**Affiliations:** 1 Division of Life Sciences and Applied Life Science (BK 21plus), College of Natural Science, Gyeongsang National University, Jinju, 52828, Korea;; 2 Department of Pharmacy, Abdul Wali Khan University Mardan, Mardan, 23200, Pakistan;; 3 Department of Pharmaceutical Chemistry, Faculty of Pharmaceutical Sciences, Riphah International University, Islamabad, Pakistan;; 4 Department of Neurology, Gyeongsang National University Hospital & College of Medicine, Gyeongsang National University, Changwon, Jinju, 52727, Republic of Korea;; 5 AI Biotics Co., Hwaseong-si, Gyeonggi-do, Republic of Korea;; 6 Department of Pharmacy, Korea University, Sejong, 20019, South Korea;; 7 Alz-Dementia Korea Co., Jinju-si, Republic of Korea

**Keywords:** Quercetin, ethanol, neurodegeneration, mitochondrial apoptotic pathway, molecular docking, therapeutic potential

## Abstract

**Introduction:**

Quercetin, a naturally occurring flavonoid, has been reported to possess several pharmacological activities including neuroprotective properties. Chronic alcohol exposure is known to cause apoptotic neurodegeneration. In this study, docking studies were used to investigate the ligand-protein affinity against various neuroinflammatory targets like ChAt, TNF, IL-6, and IL-1β. Next, molecular studies were performed to determine quercetin activity against chronic ethanol-induced neurodegeneration in the adult rat cortex.

**Methods:**

Adult rats were treated with ethanol for 3 months while quercetin was treated for the last 20 days along with ethanol to the respective experimental groups. Elements of the mitochondrial apoptotic pathway *i.e*. pro-apoptotic protein Bax, cytochrome C release, and activation of caspase-9 and caspase-3 were determined after respective drug treatment. Our docking results revealed that quercetin possesses neuroprotective potential by targeting neuroinflammatory proteins inhibiting neurodegeneration.

**Results:**

Western blot results showed that ethanol administration increased the protein expressions of Bax, cytochrome C, caspase-9, and caspase-3. Furthermore, DNA damage was also observed by chronic ethanol treatment with increased expression of PARP-1. Quercetin treatment offered neuroprotection in the cortex against ethanol-induced neurodegeneration. Quercetin reversed the ethanol-induced apoptotic trend *via* down-regulating Bax, preventing cytochrome C release and inhibition of caspase cascade.

**Conclusion:**

Immunohistological findings *i.e*. caspase-3 immunoreactivity, Nissl staining, and Fluoro-Jade B staining also revealed significant neuronal survival with quercetin treatment against ethanol-induced neuronal cell death. Our *in-silico* and *in-vivo* findings suggest that quercetin has the potential capability to be used as a neuroprotective agent against alcoholic neurotoxicity.

## 1 INTRODUCTION

Chronic ethanol ingestions have been shown to produce neurotoxicity in distinct regions of the brain which may result in several neurodegenerative disorders like Huntington’s disease, Alzheimer’s disease, or Parkinson’s disease [1-3]. Normally, ethanol causes disinhibition, euphoria, unconsciousness, sedation, confusion, incoordination, and coma at the initial stage depending upon the dose of ethanol consumed. Following 3-6 months of ethanol treatment, there appear some structural changes and regional neuronal loss in the brain [2, 4-6].

It is well known that chronic ethanol treatment is grounds for neuropathological changes in the form of apoptosis or necrosis [7, 8]. Neurological problems associated with ethanol are due to increased reactive oxygen species production which occurs from the change in metabolic processes after ethanol induction [9, 10]. Although the exact mechanism for ethanol-induced neurodegeneration is not known, studies have shown that ethanol treatment induces neuronal apoptosis *via* the generation of reactive oxygen species [11-14]. Exposure to cytotoxic agents causes the generation of death signals such as oxidative stress and DNA damage that circulates inside the cell leading to the activation of the mitochondrial apoptotic pathway [15-17]. Oxidative stress causes disturbance of mitochondrial transmembrane potential that results in the release of proapoptotic proteins like Bax, followed by cytochrome c release, and activation of caspase-9 and -3, which ultimately leads to cell death [18-21].

Recent studies have described flavonoids' beneficial role in attenuating health problems like the prevention of cardiovascular diseases, inflammatory diseases, and neurodegenerative diseases [22, 23]. Recent studies have shown that phytonutrient and their derivatives interfere with multiple signaling pathways including antioxidant defenses, protein homeostasis, natural apoptotic mechanism metabolism regulations, *etc*. [24]. As natural food derivatives, the role of flavonoids cannot be underestimated in treating neurological problems associated with alcoholism. One such compound widely found in fruits and vegetables such as onions, broccoli, berries, and apples is quercetin [25, 26]. Quercetin has been studied for several biological activities including anti-histaminic, anti-oxidant, anti-carcinogenic, and anti-inflammatory properties [27]. Quercetin is also reported to protect against oxidative injuries and cytotoxicity [28] and helps to improve learning and memory behaviors [29]. Furthermore, quercetin can penetrate the blood-brain barrier [30] and has shown neuroprotective properties against ischemia-, oxidative stress-, or neurotoxins-induced neurotoxicity [28, 31, 32]. In addition to this, *in-vitro* studies also described that quercetin effectively maintained cell survival after neurotoxic insults in different cell lines such as PC12 cells [33], C6 glioma cells [34], and SH-SY5Y human neuroblastoma cells [35].

**Fig. (1):**
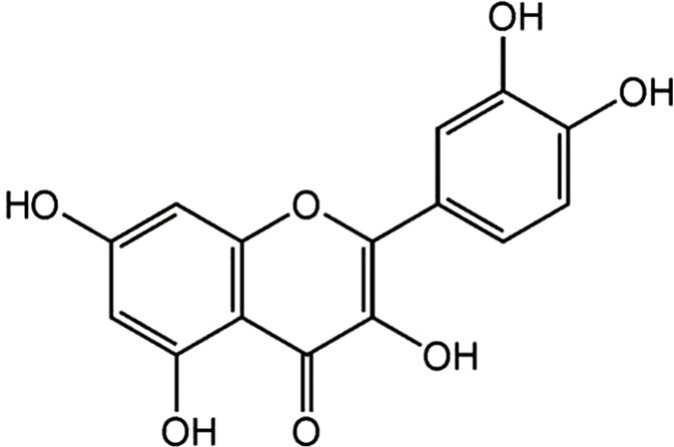
The chemical structure of quercetin.

Given its pluripotent properties, we hypothesize that quercetin, a natural flavonoid [32] can protect against ethanol-induced neurodegeneration *via* inhibiting ROS formation (Fig. **1**). In this study, we investigated the neuroprotective effect of quercetin against ethanol-induced neurodegeneration in the cortex of adult rats. The neuroprotective role of quercetin was determined by evaluating various elements of the mitochondrial apoptotic pathway. Moreover, histological studies were also performed to examine the potential effectiveness of quercetin against the neurotoxic effects of ethanol.

## 2 MATERIALS AND METHODS

### 2.1 Materials

The anti-Bax antibody, anti-cytochrome C antibody, and anti-PARP-1 antibody were purchased from Santa Cruz Biotechnology. An anti-caspase-3 antibody, anti-caspase-9 antibody, and anti-Actin antibody were brought from Cell Signaling Biotechnology. The secondary antibodies used in our experiments were goat anti-mouse IgG, goat anti-rabbit IgG, and rabbit anti-goat IgG, purchased from Santa Cruz Biotechnology. Quercetin and cresyl violet were purchased from Sigma Biotechnology.

### 2.2 Computational/Docking Studies

Docking experimental studies have been performed to discover the association of quercetin with targeted proteins/enzymes. The structures of all targeted proteins were extracted from the Protein Data Bank (http/www.rcsb.com). In analyzing the potential of a selected natural compound to cure neurodegenerative illness, choline acetyltransferase ChAt, interleukin IL-1β, interleukin IL-6, and tumor necrosis factor TNF-α are prospective targets that indicate the ability of cure. The structures of targeted proteins were downloaded as Pdb Id: 2FY2, 6Y8M, 1ALU, and 2AZ5 respectively. The procedure was performed utilizing PyRx-0.8 interlinked with Auto Dock Vina 1.1.2 latest software. The configuration of quercetin was settled in Chem draw Ultra 2.0 software in which structure have been sketched and stored in the format of mol. File. Additionally, this structure was saved in Pdb format using Discovery studio visualizer DSV. Binding energies were analyzed and effects were measured from docking experiments. The best posture of ligands interactions with proteins were obtained that gave enhanced negative value. As greater the negative, the greater will be binding affinity in that pose. The best poses were stored and visualized *via* Ligplot + software, Bio *via* Discovery Studio Visualizer, and Pymol 1.8. Various conditions were applied to visualize the amino acid residues, best interactions, bond lengths, and binding pockets in variant colors along with receptors.

### 2.3 Animal Grouping and Drug Treatment

**Fig. (2):**
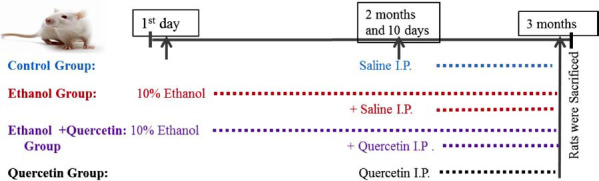
Schematic representation of animal’s treatment.

Adult male Sprague-Dawley rats (C57BL/6N N32, 8 weeks old; 25-30 g) were purchased from Samtako Biolabs (Ulsan, South Korea) (n=42), at the start of the treatment and were housed in a temperature-controlled environment and maintained on a 12 hr light/dark cycle (lights on at 6:00 am) with food *ad libitum*. A saline solution of quercetin was made in 2% dimethyl sulfoxide (DMSO). The rats were randomly divided into four groups, *i.e*. control group + saline (0.5 ml saline i.p. for last 20 days), ethanol group (10% ethanol orally for 3 months and 0.5 ml saline i.p. for last 20 days), ethanol + quercetin (10% ethanol orally for 3 months and 50 mg/kg i.p. for last 20 days) and control + quercetin (50 mg/kg i.p. for last 20 days) (Fig. **2**).

All the experimental procedures were carried out per the rules established by the animal ethics committee (IACUC) of the Division of Applied Life Sciences, Department of Biology, Gyeongsang National University South Korea.

### 2.4 Western Blot Analysis

Western blot analysis was performed according to the previously determined procedure [36]. The experimental rats were anesthetized and decapitated and the brain samples were differentiated. Cortex was removed, snap-frozen in liquid nitrogen, and stored at -80°C. All the brain tissues were homogenized in 0.2 M PBS and protein samples were collected after centrifugation. The samples were run through SDS-PAGE on 7-18% gels under reducing conditions and transferred onto a polyvinylidene difluoride (PVDF) membrane. Immunoblotting was performed using rabbit-derived Bcl-2, Bax, caspase-3, and caspase-9 antibodies or goat derived cytochrome C antibody or mouse-derived poly (ADP ribose) polymerase-1 (PARP-1) antibody (Table **1**). Anti-β-actin antibody served as a loading control. The respective membranes were probed with a goat-derived horseradish peroxidase-conjugated anti-rabbit IgG or anti-goat IgG or anti-mouse IgG secondary antibodies (Santa Cruz Biotechnology, CA, USA). Immunocomplexes were visualized using enhanced chemiluminescence (Amersham ECL Advance Western Blotting Detection reagent). The X-ray films were scanned and the optical densities of the bands were measured using computer-based Sigma Gel software (Jandel Scientific, San Rafael, Chicago, USA).

### 2.5 Tissue Collection and Sample Preparation

The experimental rats (n=6 per group) were anesthetized and sacrificed and the brain samples were removed after transcardial perfusion with ice-cold normal saline and paraformaldehyde. The brains were fixed with paraformaldehyde for s3 days and then cryoprotected with 20% sucrose phosphate buffer for 3 days. Coronal plane sections of the brain samples were thaw-mounted on the gelatin-coated slides at room temperature and stored at -70°C.

### 2.6 Immunohistochemistry

Immunohistochemistry for caspase-3 determination was performed as described by previous protocols [37]. The rat’s cortex slides were washed three times with 0.01M PBS and incubated with proteinase k for 5 min. The slides were rewashed with PBS and then quenched in a solution of methanol containing 3% hydrogen peroxide for 10-15 min. Tissue slides were washed twice with PBS, followed by incubation for 1hr in blocking solution (5% normal goat serum), and then incubated overnight in blocking solution consisting of primary antibody, caspase-3 (rabbit 1:100; cell signaling). The slides were washed 3 times with PBS and then secondary antibodies (1:500 in PBS) were applied. Slides were then rewashed with PBS, followed by treatment with ABC reagent for 60 min. The tissue sections were then treated with DAB reagent until a light brownish color appeared on the tissue sections. The slides were washed with PBS, and immersed in xylene, and glass coverslips were mounted on glass slides with a mounting medium. The images were captured with a fluorescent light microscope.

### 2.7 Fluoro-Jade B (FJB) Staining

Fluoro-Jade B staining was performed as described by the previous protocol [38]. Tissue sections were air-dried overnight, washed with PBS, and immersed in a solution of 1% sodium hydroxide and 80% ethanol for 5 mins. The tissue sections were then treated with 70% alcohol followed by a solution of 0.06% potassium permanganate for 10 min. Sample slides were then rinsed with distilled water and immersed in a solution of 0.1% acetic acid and 0.01% Fluoro-jade B for 20 mins. Finally, the slides were washed with distilled water three times for 1 minute each and allowed to dry at a warm temperature for 10 mins. Glass coverslips were mounted using DPX non-fluorescent mounting medium, and images were prepared with confocal laser scanning microscope (FV 100, Olympus, Japan). The numbers of active neuronal cells in the cortex sections were counted using a computer-based image J program analysis.

### 2.8 Cresyl Violet Staining of Nissl Bodies

Cresyl violet staining was performed by the same method as described in previous studies with some modifications [39]. Brain tissue slides were dried overnight and washed twice with 0.01 M PBS for 10 min each. The staining solution was prepared by dissolving 0.5% Cresyl violet acetate (Sigma) in distilled water, filtering and adding a few drops of glacial acetic acid to the solution. The tissue sections were stained with cresyl violet solution for 10-15 mins, rinsed with distilled water, and dehydrated in graded alcohol. Finally, the slides were immersed in xylene for 5 mins, glass coverslips were mounted on the slides, and images were prepared with a fluorescent light microscope.

### 2.9 Data Analysis

Statistical analyses were performed using two-way analysis of variance (ANOVA) for repeated measures. After ANOVA analysis, post hoc pair-by-pair differences between groups were determined using the Boneforoni multiple comparison test. Calculations and graphical presentation were performed with the statistical software Graph Pad Prism 10.

## 3 RESULTS

### 3.1 Docking Studies

To analyze the pharmacological performance of Quercetin with ethanol-induced neurodegenerative models, certain proteins were targeted that provide evidence of interaction of Quercetin from the perception of enzyme-ligand interactions, docking studies were performed using the Pdb Ids of all earlier described crystal assembly of ChAt, TNF-α, IL-6, IL-1β as macromolecular models. These best docking postures were scrutinized to interpret different interaction parameters. The results of the interaction have been tabulated in Table **1**.

Quercetin when docked with choline acetyltransferase ChAt (Pdb Id: 2FY2) gave good binding affinities with values of -7.3 Kcal/mol. it formed one conventional hydrogen bond with Gln 541 at a bond length of 1.95Å. Further, it gave two *pi-pi* stacked bonds with Tyr 552. One *pi*-donor hydrogen was observed with Tyr 436 at a bond length of 3.14 Å. The interactions are shown in Fig. (**3**).

Tumor Necrosis factor (TNF-α) plays a critical role in promoting apoptosis that leads to the production of pro-inflammatory cytokines that ultimately cause neuronal inflammation and death. The exposure to ethanol may cause neurodegeneration and this protein may be involved in this process. The Quercetin was analyzed for interaction with TNF-α with Pdb Id: 2AZ5 that showed promising binding affinities with an energy of -7.6 Kcal/mol. This binding was supported with two conventional hydrogen bonds, formed with Gly 121 and Leu 120 at the bond length of 2.41 Å and 2.74 Å respectively. Further, one conventional hydrogen bond was observed with Tyr 151 at the bond length of 2.81 Å. *Pi-pi* stacked interactions were seen with Tyr 59. Other amino acid residues include Gln 61, Tyr 119, Gly 121, Tyr 59, Ser 60, and Ile 155 accordingly. All the interactions have been shown in Fig. (**4**).

In analyzing the effects of Quercetin with neurodegenerative animal models, interleukin proteins have its role. Quercetin gave -6.8 Kcal/mol as binding affinity with interleukin-6 IL-6 (Pdb Id: 1ALU). The amino acid residues for receptors involved Asn 63, Leu 62, and Pro 139. One strong conventional bond was observed with Asn 144 with a bond length of 2.28 Å. Pi-pi stacked bond was observed with Tyr 97 and the pi-sigma bond was seen with Leu 147 at the bond length of 3.72 Å. Further carbon-hydrogen bond was visualized as having bod length of 3.68 Å. All interactions have been depicted in Fig. (**5**).

The molecular docking studies provide convincing evidence that supports the inhibitory potential of Quercetin to recover the neurodegenerative behavior of animal models. Quercetin was analyzed with interleukin-1β (Pdb Id: 6Y8M) that gave binding energies of -7.1 Kcal/mol inside the binding pocket of the protein. It gave four conventional hydrogen bonds with amino acid residue Ser 43, Lys 65, Tyr 68, and Pro 87 at the bond length of 3.69 Å, 2.05 Å, 2.70 Å, and 2.16 Å respectively. It also gave a *pi*-sigma bond with Pro 91 at a bond length of 3.52 Å. Other amino acid residues were Ser 5, Asn 7, Ser 45, Gly 61, Lys 63, Gly 64, Asn 66, Leu 67, Lys 88, and Tyr 90. All results are shown in Fig. (**6**).

### 3.2 Effect of Quercetin on Ethanol-induced Expressions of Bax and Cytochrome C Proteins in the Cortex of Adult Rats

**Fig. (7):**
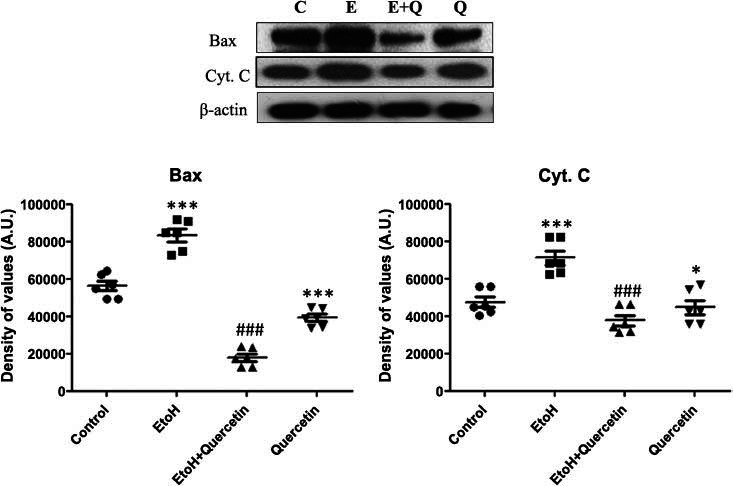
Effect of quercetin on ethanol-induced expression of Bax and cytochrome C levels of proteins. Representative western blots showed a level of proteins probed with antibodies of Bax and cytochrome C after respective treatment with ethanol (2 gm/kg) and quercetin (50 mg/kg), in the cortex of adult rats. Sigma gel software was used to quantify the protein bands and the density values are expressed in arbitrary units for all the proteins (n=6 animals per group). ***p< 0.0001, *p< 0.05 *versus* control group. ###*p*< 0.0001 *versus* ethanol-treated group.

Bax is a proapoptotic member of the Bcl-2 family of proteins. Previous research studies have shown that quercetin prevents cellular apoptosis *via* upregulation of the pro-apoptotic proteins [40]. Our result showed that ethanol treatment significantly elevated the level of Bax expression as compared to the control group (*p* > 0.0001), while quercetin treatment significantly decreased the elevated level of Bax, compared to the ethanol-treated group, (F (3,15) = 706.6 ; *p* > 0.0001) (Fig. **7**).

Activation of Bax leads to the release of cytochrome C, an inner mitochondrial membrane protein, from mitochondria into the cytosol which leads to activation of caspase cascade [41]. Therefore, we measured the level of cytochrome C to determine the effect of ethanol and ethanol plus quercetin in the cortical part of the brain. Western blot results revealed that chronic ethanol treatment significantly increased the expression of cytosolic cytochrome C compared to the control group (*p* > 0.0001). Interestingly, rats treated with ethanol along with quercetin showed a significant reduction in the expression of cytochrome C relative to the ethanol-treated group, (F (3,15) = 626.2 ; *p* > 0.0001) (Fig. **7**).

### 3.3 Effect of Quercetin on the Ethanol-induced Activated Expression of Caspase-9, Caspase-3, and PARP-1 Proteins

**Fig. (8):**
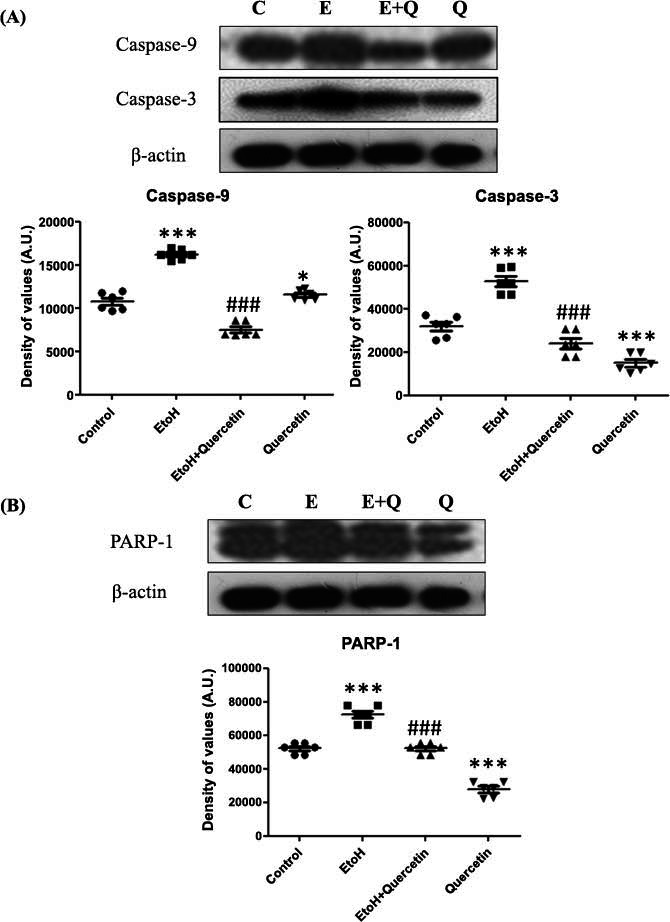
(**A**) Effect of quercetin on ethanol-induced expression of caspase-3 and caspase-9 levels of proteins. Representative western blots showed a level of proteins probed with antibodies of caspase-9 and caspase-3 after respective treatment with ethanol (2 gm/kg) and quercetin (50 mg/kg), in the cortex of adult rats. Sigma gel software was used to quantify the protein bands and the density values are expressed in arbitrary units for all the proteins (n=6 animals per group). ***p< 0.0001, *p< 0.05 *versus* control group. ###*p*< 0.0001 *versus* ethanol-treated group. (**B**) Effect of quercetin on ethanol-induced expression of PARP-1 levels of proteins. Representative western blot showed the level of proteins probed with antibodies of PARP-1 after respective treatment with ethanol (2 gm/kg) and quercetin (50 mg/kg), in the cortex of adult rats. Sigma gel software was used to quantify the protein bands and the density values are expressed in arbitrary units for all the proteins (n=6 animals per group). ***p< 0.0001 *versus* the control group. ###*p*< 0.0001 *versus* ethanol-treated group.

To investigate the effective inhibition of caspases at the level of downstream apoptotic pathways by quercetin, western blot analyses were performed. Western blot results showed that ethanol administration significantly increased the expression of caspase-9 and caspase-3 compared to the control group (*p* > 0.0001), while quercetin treatment along with ethanol significantly decreased the level of activated caspase-9 and caspase-3, compare to the ethanol-treated group in the cortex of adult rats (caspase-9: F (3,15) = 427.8; caspase-3: (F (3,15) = 452.6; *p* > 0.0001) (Fig. **8A**).

PARP-1 is one of the hallmarks of apoptosis is also observed in this study. To elucidate the level of DNA damage by ethanol treatment, PARP-1 activation was determined by western blot analysis. Our results showed that ethanol treatment significantly elevated the level of PARP-1 expression in the cortex of the ethanol-treated group as compared to the saline-treated group (*p* > 0.0001). Interestingly, rats treated with ethanol along with quercetin showed a significant reduction in the level of cleaved PARP-1 as compared to the ethanol-treated group, (F (3,15) = 1868; *p* > 0.0001) (Fig. **8B**).

### 3.4 Effect of Quercetin on the Ethanol-induced Immunohistological Expression of Caspase-3

**Fig. (9):**
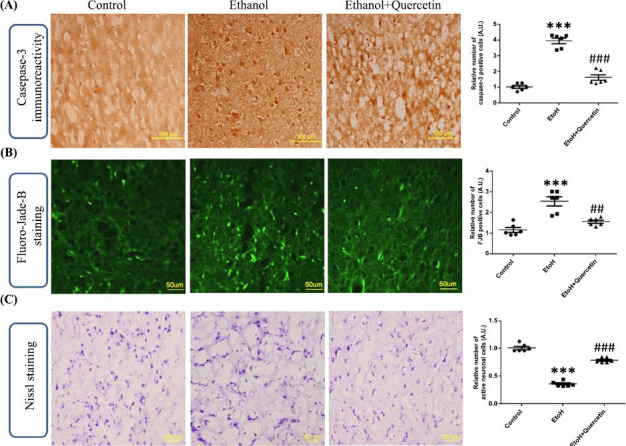
(**A**) Representative photomicrographs of immunohistochemical analysis of activated caspase-3 positive cells in the experimental groups. All the panels representing cortex showed caspase-3-stained brain tissue at magnification 20x objective field, scale bar = 100 µm. (**B**) Shown are representative photomicrographs of FJB staining (magnification 40x objective field, scale bar = 50 µm) and (**C**) Nissl staining (magnification 20x objective field, scale bar = 200 µm), for dead and damaged neurons. Images are representative of staining obtained in sections prepared from at least 6 animals per group. ***p< 0.0001, ###*p*< 0.001, ##*p*< 0.0001 *versus* ethanol treated group.

The western blot results of apoptotic pathway were supported by immunohistochemical analysis, where we confirmed the role of quercetin for caspase-cascade inhibition. Expressions of caspase-3 immunoreactive cells were analyzed in control and experimental groups which showed that ethanol treatment significantly enhances caspase-3 expression in the cortex as compared to the control group. Treatment with ethanol along with quercetin showed a significant reduction in the number of caspase-3 active cells as compared to the ethanol-treated group (F (2, 15) = 262.5; *p* > 0.0001) (Fig. **9A**).

### 3.5 Effect of Quercetin on Ethanol-induced Neurodegeneration

Being a reliable marker for the detection of dead or damaged neuronal cells [42], FJB staining was performed to detect the extent of neuronal survival in ethanol and ethanol plus quercetin treated rats. Following the FJB staining procedure, immunofluorescence declared several FJB positive neuronal cells (damaged neurons) in the cortex of the ethanol-treated group relative to the control group. Our results showed that administration of quercetin along with ethanol significantly inhibit ethanol-induced neuronal damage and retained the neuronal viability compared to the ethanol-treated group, as indicated by a decreased number of FJB positive cells (F (2, 15) = 30.6; *p* > 0.0001) (Fig. **9B**).

Nissl staining was performed to find out the extent of neuronal viability after chronic ethanol exposure either alone or in combination with quercetin. Ethanol-treated rats showed several damaged, fragmented and shrunk neuronal cells with the least number of normal neurons as compared to the control group. The numbers of active neurons in the ethanol-treated group were found significantly less than that of the control group. Treatment of quercetin along with ethanol resulted in a significantly increased level of active neurons as compared to the ethanol-treated group (F (2, 15) = 359.9; *p* > 0.0001) (Fig. **9C**). Overall, these results showed that quercetin treatment effectively inhibits ethanol-induced neurodegeneration in the cortex of adult rats.

## 4 DISCUSSION

Programmed cell death or cellular apoptosis involves several morphological or biochemical changes that affect normal cellular physiology. These changes include mitochondrial depolarization, nuclear fragmentation, membrane blister formation, cell shrinkage, and the formation of apoptotic bodies. Neuronal apoptosis in characteristic regions of the brain is involved in the progression of different neurodegenerative diseases like Alzheimer’s disease, Parkinson’s disease, and Huntington’s disease [43]. Earlier studies have shown that chronic ethanol treatment induces several neurotoxic and cytotoxic effects that cause structural and functional disturbances in the brain. These neurodegenerative effects of long-term alcoholism are associated with a wide range of neurological deficits including cognitive and memory impairments. Ethanol administration causes the induction of oxidative stress that leads to the activation of neuronal apoptotic pathways *via* the activation of caspase cascade in the cytosol [12, 44, 45]. Several approaches have been described to elucidate the molecular mechanism for ethanol-induced neurodegeneration which include the inhibition of N-methyl-D aspartate receptors [46, 47], generation of reactive oxygen species [48-50], increase in super anion production *via* disturbing the mitochondrial electron transport chain [48], and a hyperexcitability state with ethanol withdrawal [2]. Other research bodies have reported that alcohol administration induces neurotoxicity *via* disruption of cell-cell interactions, accumulation of ROS and RNS, and obstruction in the normal functioning of growth factors in the brain [49]. Several studies have shown the neuroprotective potential of natural products or pharmacological compounds against neurotoxicity induced by oxidative and nitrosative stress [28, 50]. In this study, we investigated the neuroprotective effect of quercetin against a neurodegenerative model of ethanol. Docking studies were performed using the Pdb Ids of all earlier described crystal assemblies of ChAt, TNF-α, IL-6, IL-1β as macromolecular models whereby our results showed that quercetin possesses strong binding activity towards these neuroinflammatory mediators. It is well established that the mitochondria-dependent apoptotic pathway involves ethanol-induced neurotoxicity. The Bcl-2 family of proteins is known to have an important role in intracellular apoptotic signal transduction.

Research studies have shown that oxidative stress activates the transcription factor NFκB, which in turn induces the assertion of defensive mitochondrial proteins like SOD2 and Bcl-2 [51]. During the early steps of apoptosis, Bax proteins act at the mitochondrial outer membrane, causing mitochondrial permeability transition pores to open and release cytochrome C into the cytosol [40]. Oxidative stress mainly targets mitochondria as it is the major intracellular source of ROS. An increase in ROS concentration leads to a disturbance of mitochondrial membrane permeability, causing its depolarization, followed by the release of cytochrome C. The activation of the caspase cascade is considered an essential step in the mitochondrial apoptotic pathway. Caspase 9 and caspase 3 are important members of the caspase cascade that are alternatively activated after the release of cytochrome C into the cytosol [37, 41]. In accordance, our results showed that chronic administration of 2 g/kg of ethanol caused neuronal cell death in the cortex of the adult rat brain. The neuronal death was determined by the formation of apoptotic bodies, activation of caspase cascade, and nuclear fragmentation. We reported that ethanol treatment significantly elevated the level of different apoptotic markers like Bax, cytochrome C, caspase-9, and caspase-3. It has also been studied that cleavages of other proteins like PARP-1 also occur upon activation of caspase-3. PARP-1 plays a key role in DNA repair in normal physiologic conditions, hence maintaining cell integrity [52]. Studies have shown that PARP-1 over-expression is responsible for neuronal cell death in the state of oxidative stress and excitotoxicity [53, 54]. During stress conditions activation of PARP-1 depletes intracellular NAD^+^ that leads to depletion of ATP and ultimately causes apoptosis or necrosis [55]. We reported that long-term ethanol treatment induces elevated expression of PARP-1 that may be responsible for DNA damage and induction of apoptosis.

Quercetin is an important compound of the flavonoid family, mostly found in fruits, vegetables, and beverages, and is known to possess antioxidative and cognitive properties [56]. Moreover, it is reported that quercetin activates the Nrf2-ARE pathway (nuclear erythroid 2-related factor-2/ antioxidant response element pathway) which may be responsible for the antioxidant role of quercetin [57]. It has been stated that oral or I/V administration of quercetin up to 56 mg/kg body weight in rats, did not cause any significant adverse effects [58]. In this study, we demonstrated that quercetin at a dose of 50 mg/kg attenuated the neurotoxic effect of ethanol *via* inhibiting mitochondrial apoptotic cascade. Bax, an anti-apoptotic protein is one of the primary proteins involved in the activation of the apoptotic pathway after mitochondrial membrane depolarization [59, 60]. Earlier studies have shown that Bax channel inhibitors effectively inhibit cytochrome C efflux from brain mitochondria which further obstruct the caspase cascade and relieve apoptosis [61, 62]. We found that quercetin effectively inhibits the release of apoptotic markers like Bax, cytochrome C, caspase 9, and caspase 3, and alleviates DNA damage by maintaining PARP-1 activity.

The immunohistochemical findings of this study showed similar results with the western blot findings. Both molecular and morphological results showed an increase in apoptotic cell death with ethanol treatment. Nissl and FJB staining showed several damaged or dead neuronal cells in the cortex of ethanol-treated rats following quercetin treatment, effective inhibition of the neuronal damaged or dead cells is observed. Several lines of evidence have confirmed that quercetin can cross the blood-brain barrier [30] and effectively scavenge free radicals [63], therefore it can be hypothesized that the free radical scavenging property of quercetin may be responsible for its neuroprotective effect against ethanol-induced neurotoxicity.

## CONCLUSION

In conclusion, our results showed that quercetin has binding affinities against different targets involved in neuroprotection. Quercetin attenuates protein elements of mitochondrial apoptotic pathways that were increased by ethanol induction. Overall, quercetin has effective therapeutic potential to attenuate ethanol-induced neurotoxicity in the adult rat’s brain. We suggest further research studies to elucidate the exact molecular mechanism for the neuroprotective property of quercetin.

## Figures and Tables

**Fig. (3):**
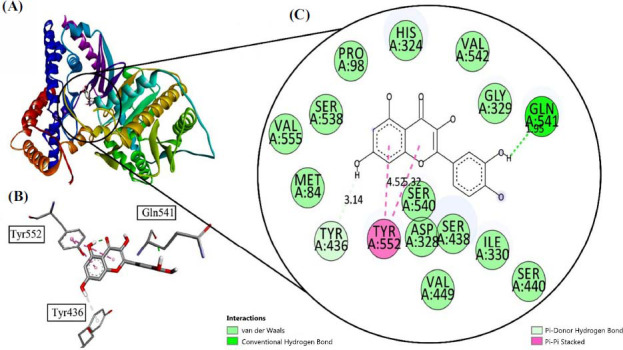
(**A**) Visualization of Quercetin inside the choline acetyltransferase (Pdb Id: 2FY2), (**B**) interaction with receptor active side, (**C**) two-dimensional view inside active side with amino acid residues shown.

**Fig. (4):**
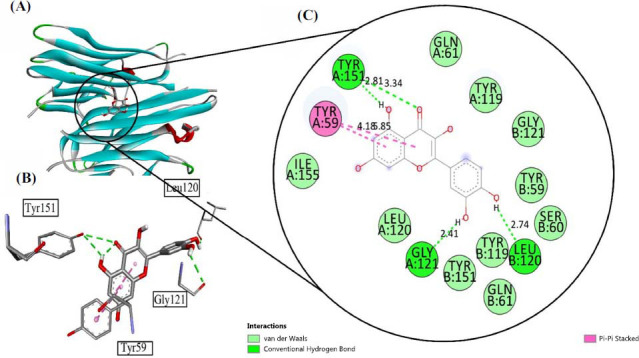
(**A**) Visualization of Quercetin inside the Tumor Necrosis Factor (Pdb Id: 2FY2), (**B**) interaction with receptor active side, (**C**) two-dimensional view inside active side with amino acid residues shown.

**Fig. (5):**
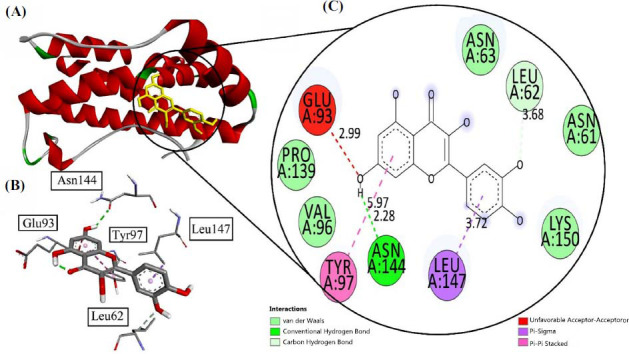
(**A**) Visualization of Quercetin inside the interleukin-6 (Pdb Id: 1ALU), (**B**) interaction with receptor active side, (**C**) two-dimensional view inside active side with amino acid residues shown.

**Fig. (6):**
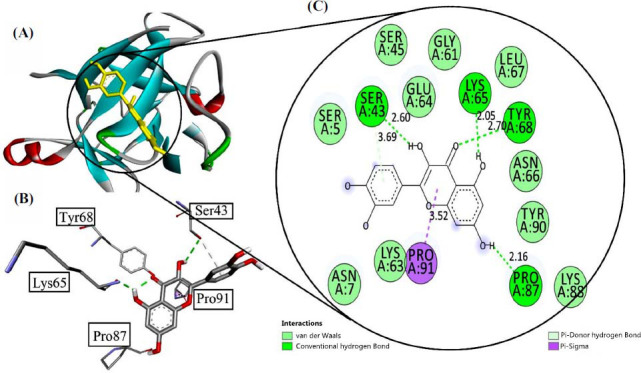
(**A**) Visualization of Quercetin inside the interleukin 1-1β (Pdb Id: 6Y8M), (**B**) interaction with receptor active side, (**C**) two-dimensional view inside active side with amino acid residues shown.

**Table 1:** Docking Score and Root Mean Square Deviation (RMSD) values of targeted protein.

**S. No.**	**Proteins**	**Pdb Id**	**Binding Energy Kcal/Mol**	**RMSD Lower Bond**	**RMSD Upper Bond**
1	ChAt	2FY2	-7.3	15.255	17.466
2	TNF-α	2AZ5	-7.6	4.251	5.495
3	IL-6	1ALU	-6.8	27.296	29.324
4	IL-1β	6Y8M	-7.1	25.958	28.239

## Data Availability

The data and supportive information are available within the article.

## LIST OF ABBREVIATIONS

PARP-1: Polymerase-1
PVDF: Polyvinylidene Difluoride
TNF-α: Tumor Necrosis Factor
